# Tapered vs. Uniform Tube-Load Modeling of Blood Pressure Wave Propagation in Human Aorta

**DOI:** 10.3389/fphys.2019.00974

**Published:** 2019-08-06

**Authors:** Azin Mousavi, Ali Tivay, Barry Finegan, Michael Sean McMurtry, Ramakrishna Mukkamala, Jin-Oh Hahn

**Affiliations:** ^1^Department of Mechanical Engineering, University of Maryland, College Park, MD, United States; ^2^Department of Anesthesiology and Pain Medicine, University of Alberta, Edmonton, AB, Canada; ^3^Department of Medicine, University of Alberta, Edmonton, AB, Canada; ^4^Department of Electrical and Computer Engineering, Michigan State University, East Lansing, MI, United States

**Keywords:** tube-load model, aorta, blood pressure, wave propagation, aortic tapering, pulse transit time, reflection constant

## Abstract

In this paper, tapered vs. uniform tube-load models are comparatively investigated as mathematical representation for blood pressure (BP) wave propagation in human aorta. The relationship between the aortic inlet and outlet BP waves was formulated based on the exponentially tapered and uniform tube-load models. Then, the validity of the two tube-load models was comparatively investigated by fitting them to the experimental aortic and femoral BP waveform signals collected from 13 coronary artery bypass graft surgery patients. The two tube-load models showed comparable goodness of fit: (i) the root-mean-squared error (RMSE) was 3.3+/−1.1 mmHg in the tapered tube-load model and 3.4+/−1.1 mmHg in the uniform tube-load model; and (ii) the correlation was *r* = 0.98+/−0.02 in the tapered tube-load model and *r* = 0.98+/−0.01 mmHg in the uniform tube-load model. They also exhibited frequency responses comparable to the non-parametric frequency response derived from the aortic and femoral BP waveforms in most patients. Hence, the uniform tube-load model was superior to its tapered counterpart in terms of the Akaike Information Criterion (AIC). In general, the tapered tube-load model yielded the degree of tapering smaller than what is physiologically relevant: the aortic inlet-outlet radius ratio was estimated as 1.5 on the average, which was smaller than the anatomically plausible typical radius ratio of 3.5 between the ascending aorta and femoral artery. When the tapering ratio was restricted to the vicinity of the anatomically plausible typical value, the exponentially tapered tube-load model tended to underperform the uniform tube-load model (RMSE: 3.9+/−1.1 mmHg; *r* = 0.97+/−0.02). It was concluded that the uniform tube-load model may be more robust and thus preferred as the representation for BP wave propagation in human aorta; compared to the uniform tube-load model, the exponentially tapered tube-load model may not provide valid physiological insight on the aortic tapering, and its efficacy on the goodness of fit may be only marginal.

## Introduction

Cardiovascular disease (CVD) is the leading cause of mortality and morbidity that imposes profound impact on health and economy in the United States as well as globally (Benjamin et al., 2018). According to the recent statistics reported by the American Heart Association, CVD is currently responsible for more deaths each year than cancer and chronic lower respiratory disease (which are the second and third cause of death, respectively) combined in the United States. In addition, CVD represents a considerable economic burden to the society: by 2035, 45.1% of the population in the United States would have some form of CVD and total costs of CVD are expected to reach $1.1 trillion. In 2014, a total number of 7.4 million cardiovascular (CV) surgical procedures were performed in the United States, which was one of the two most frequently performed surgical procedures.

To date, non-invasive brachial arterial blood pressure (BP) measured by the auscultation technique remains the mainstay of CV health and disease assessment (Black et al., 1997; Miura et al., 2001; Amar, 2002; Lewington et al., 2002; Haider et al., 2003; Turnbull et al., 2003; Palmieri et al., 2006). However, recent work has suggested that central aortic BP measured in the vicinity of the heart may serve as superior signature of CV health and disease to the conventional brachial BP (Safar et al., 2002; Roman et al., 2007, 2010; Ferguson et al., 2008; Jankowski et al., 2008; Pini et al., 2008; Vlachopoulos et al., 2010; McEniery et al., 2014; Ochoa et al., 2018). Regardless, the widespread use of central aortic BP for CV health and disease assessment has been largely hampered by the challenges associated with its direct measurement, including the requirement for inconvenient and risky clinical procedures as well as trained operators [e.g., cardiac catheterization (Sharman et al., 2006; Ding et al., 2011; Fazeli et al., 2014; McEniery et al., 2014) and carotid artery tonometry (Chen et al., 1996;Gallagher et al., 2004)].

To exploit the superior clinical value of the central aortic BP while still leveraging the convenience of distal (e.g., brachial) BP measurement, many attempts have been made to derive central aortic BP from distal BP measurement(s). Currently prevalent approach is a population-based, frequency-domain transformation known as the Generalized Transfer Function (GTF), which converts a distal [e.g., brachial (Sharman et al., 2006; Cheng et al., 2012; Shih et al., 2013) or radial (Chen et al., 1997; Cameron et al., 1998; Hope et al., 2007)] arterial pulse waveform into central aortic BP waveform. While demonstrated to be effective in some prior work, the GTF technique has a critical shortcoming: the GTF is not patient-specific by nature, whereas the characteristics associated with the propagation of BP waves in the arteries exhibit a large degree of inter- and intra-individual variability (Rashedi et al., 2013). Hence, the efficacy of the GTF technique can be variable (Hope et al., 2003). Individualizing the GTF can present significant challenges, since its expression involves a great number of parameters associated with the magnitude and phase responses at multiple frequencies.

Tube-load (TL) model has the potential to serve as an alternative to the frequency response model used in the GTF technique as well as to offer new opportunities toward patient-specific assessment of CV health by virtue of its two unique strengths: (i) it is characterized by a small number of parameters as opposed to the frequency response model, thus facilitating individualization with small amount of data; and (ii) all its parameters are equipped with physiological implications, thus facilitating the assessment of patient-specific CV health based on the individualized TL model parameters in conjunction with the BP waveform data. In fact, when combined with techniques for estimating subject-specific TL model parameters, the TL model has been shown to be very useful in estimating and monitoring arterial hemodynamic indices (Zhang et al., 2011). The most well-known TL model is the uniform lossless TL model (Zhang et al., 2011), in which an artery of interest is modeled as a uniform lossless tube (or transmission line) terminated with a load that is in general frequency dependent. In conjunction with the advances in novel techniques for estimating subject-specific parameters therein [e.g. (i) by fitting the TL model to diametric (such as arm and leg) BP waveforms and exploiting the fact that both the diametric BP waveforms originate from the unknown yet common central BP (Swamy et al., 2007; Hahn et al., 2009), or (ii) by fitting it to a distal BP waveform with physiologically relevant constraints that central blood flow is zero during diastole and/or central BP is smoother than distal BP (Swamy et al., 2009; Hahn et al., 2012; Hahn, 2014)], the uniform lossless TL model has been shown to be effective and robust in providing valuable insights related to the CV hemodynamics (Sipkema and Westerhof, 1975; Shroff et al., 1995; Burattini and Campbell, 2000; Westerhof et al., 2008; Swamy et al., 2010; Fazeli et al., 2014; Gao et al., 2014; Kim et al., 2015; Ghasemi et al., 2018).

Despite its success thus far, the simplicity of the uniform lossless TL model motivates investigations for its potential improvement by incorporating more realistic components. In particular, arteries exhibit tapering, bifurcations, and BP loss. In a series of our prior work, we investigated the TL models equipped with bifurcations and pressure loss to demonstrate that such extension of the uniform lossless TL model may lead to small but statistically significant improvement in its goodness of fit. As a follow-up work, the goal of this study was to investigate if there is any benefit in incorporating the geometric tapering into the TL model. To achieve the goal, this study comparatively investigated the tapered vs. uniform TL models as mathematical representation for BP wave propagation in human aorta. The relationship between the aortic inlet and outlet BP waves was formulated based on the exponentially tapered and uniform TL models. Then, the validity of the two TL models was comparatively investigated by fitting them to the experimental aortic and femoral BP waveform signals collected from 13 coronary artery bypass graft surgery patients.

This paper is organized as follows. Experimental data, the TL models, and the data analysis details are given in section Methods. Section Results presents key results, which are interpreted and discussed in section Discussion. Section Conclusion provides conclusions derived from the study.

## Methods

### Experimental Data

The experimental data collected in our prior work (Rashedi et al., 2013; Fazeli et al., 2014) were used. In brief, invasive central aortic and femoral BP data were collected from 13 patients undergoing coronary artery bypass graft with cardiopulmonary bypass. Inclusion criteria were: (i) ages 18–80 years, inclusive, and (ii) scheduled for coronary artery bypass graft procedure with cardiopulmonary bypass. Exclusion criteria were: (i) scheduled for heart surgery other than coronary artery bypass graft which can incur distortion of central aortic BP waveform (e.g., aortic valve repair, etc.), (ii) female of childbearing potential, (iii) emergency surgery, and (iv) body mass index >35. The study was carried out in accordance with the recommendations of the University of Alberta Health Research Ethics Board with written informed consent from all subjects. All subjects gave written informed consent in accordance with the Declaration of Helsinki. The protocol was approved by the University of Alberta Health Research Ethics Board (ID Pro00021889).

Data used in this work were collected right before or after the cardiopulmonary bypass. Following the induction of anesthesia and before the cardiopulmonary bypass, a catheter was inserted into the femoral artery. Then, a cannula was inserted into the ascending aorta by a surgeon immediately before or after the cardiopulmonary bypass. Then, ascending aortic and femoral arterial BP waveforms were recorded at a sampling rate of 1 kHz for up to 2 min.

### Tube-Load Models

**Figure 1:**
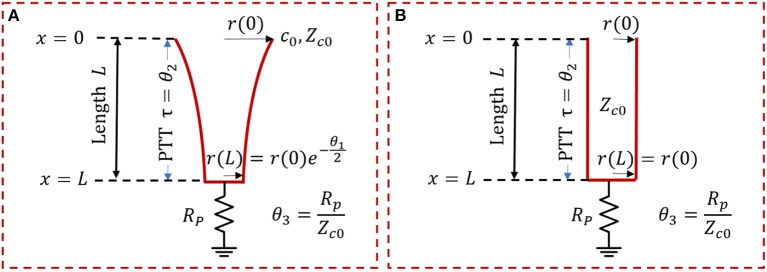
Exponentially tapered **(A)** vs. uniform **(B)** tube-load (TL) models. Exponentially tapered TL model is characterized by 3 parameters: θ_1_ = *qL*, $\theta_{2}=\tau =L\sqrt{l_{0}c_{0}}$, $\theta_{3}=\frac{R_{p}}{Z_{c0}}$, where *q* denotes the rate of tube tapering; *L* denotes the tube length; τ denotes the pulse transit time (PTT); *l*_0_, and *c*_0_ denote the inertance and compliance per unit length at the tube inlet; and *R*_*p*_ and *Z*_*c*0_ denote the terminal load resistance and tube characteristic impedance at the tube inlet, respectively. Uniform TL model is derived as a special case of exponentially tapered TL model when *q* = 0 and is thus characterized by 2 parameters θ_2_ and θ_3_.

In this study, a variant of the exponentially tapered TL model of the aorta developed by Fogliardi et al. (1997) and a uniform TL model were investigated (Figure 1). The tapered TL model relates the aortic inlet (*P*(*jω*, 0)) and outlet (*P*(*jω, L*)) pressures by way of the following transfer function (see Appendix for details):

(1) $$\begin{array}{rcl} \frac{P\left(j\omega ,L\right)}{P\left(j\omega ,0\right)} & = & H\left(j\omega |\theta_{1},\theta_{2},\theta_{3}\right)\\ = & \frac{2\theta_{3}\bar{\Delta }e^{-\left[\bar{\Delta }-\frac{\theta_{1}}{2}\right]}}{\theta_{3}\left(\bar{\Delta }+\frac{\theta_{1}}{2}\right)+j\omega \theta_{2}e^{\theta_{1}}+\left[\theta_{3}\left(\bar{\Delta }-\frac{\theta_{1}}{2}\right)-j\omega \theta_{2}e^{\theta_{1}}\right]e^{-2\bar{\Delta }}} \end{array}$$

where θ_1_ = *qL*, $\theta_{2}=\tau =L\sqrt{l_{0}c_{0}}$, $\theta_{3}=\frac{R_{p}}{Z_{c0}}$, and $\bar{\Delta }=\bar{\Delta }\left(\theta_{1},\theta_{2}\right)=\sqrt{{\left(\frac{\theta_{1}}{2}\right)}^{2}+{\left(j\omega \theta_{2}\right)}^{2}}$. The parameters are equipped with the following physical meanings: (i) *q* denotes the rate of tube tapering; (ii) *L* denotes the tube length; (iii) τ denotes the pulse transit time (PTT), time taken for the BP wave to travel from the inlet to the outlet of the aorta (Mukkamala et al., 2015); (iv) *l*_0_ and *c*_0_ denote the inertance and compliance per unit length at the tube inlet; and (v) *R*_*p*_ and *Z*_*c*0_ denote the terminal load resistance and tube characteristic impedance at the tube inlet, respectively. The uniform TL model is derived as a simplified case of the tapered TL model when *q* = 0 (meaning that there is no tapering). Given that θ_1_ = 0 and $\bar{\Delta }=j\omega \tau$ when *q* = 0, Equation (1) reduces to the following:

(2) $$\begin{array}{rcl} \frac{P\left(j\omega ,L\right)}{P\left(j\omega ,0\right)} & = & H\left(j\omega |\theta_{1}=0,\theta_{2},\theta_{3}\right)\\ = & \frac{2\theta_{3}}{\left(\theta_{3}+1\right)e^{j\omega \theta_{2}}+\left(\theta_{3}-1\right)e^{-j\omega \theta_{2}}} \end{array}$$

It is noted that θ_1_ implies the absolute extent of tapering between the tube inlet and outlet cross-sectional radii: $\frac{r\left(L\right)}{r\left(0\right)}=e^{-\frac{\theta_{1}}{2}}$. In addition, the reflection constant at the tube-load interface is given by $\Gamma =\frac{\theta_{3}-1}{\theta_{3}+1}$.

### Data Analysis

The validity of the tapered and uniform TL models was investigated and compared by fitting the models to the ascending aortic and femoral arterial BP waveforms associated with each subject on an individual basis. Details follow.

In each subject, a 15 beat-long pair of ascending aortic and femoral arterial BP waveforms were extracted from the recorded data and then down-sampled at 100 Hz. The first 10 beat-long data (called the training data) were used for model fitting, while the remaining 5 beat-long data (called the testing data) were used for assessing the validity of the models thus fitted. In this way, the TL models could be tested in the same CV state as when they were trained using the data not presented in the training process. For the sake of model fitting, the following optimization problem was solved using MATLAB and its Optimization Toolbox in order to derive the optimal parameter estimates θ^*^ associated with each subject from the training data:

(3) $$\begin{array}{r} \theta^{*}=\arg\underset{\theta \in \Omega_{\theta }}{\text{min}}||P\left(t,L\right)-\hat{P}\left(t,L|\theta \right)|| \end{array}$$

where *P*(*t, L*) is the aortic outlet BP (i.e., the femoral arterial BP) at time *t*, and $\hat{P}\left(t,L|\theta \right)$ is the aortic outlet BP at time *t* predicted by the (tapered or uniform) TL model equipped with the parameters θ = {θ_1_, θ_2_, θ_3_}, when the aortic inlet BP (i.e., the ascending aortic BP) data were inputted. The domain Ω_θ_ was defined as Ω_θ_ = {θ|θ_1_ > 0, θ_2_ > 0, θ_3_ > 0} based on the physical meanings of the TL model parameters. The model-predicted aortic outlet BP $\hat{P}\left(t,L|\theta \right)$ was computed as follows. Given the set of TL model parameters θ at each iteration of optimization, the aortic inlet BP signal *P*(*t*, 0) in the time domain was transformed via the fast Fourier transform (FFT) to yield the frequency-domain signal *P*(*jω*, 0). Then, the frequency-domain aortic outlet BP signal $\hat{P}\left(j\omega ,L|\theta \right)$ was computed as follows:

(4) $$\begin{array}{r} \hat{P}\left(j\omega ,L|\theta \right)=H\left(j\omega |\theta_{1},\theta_{2},\theta_{3}\right)P\left(j\omega ,0\right) \end{array}$$

Finally, $\hat{P}\left(j\omega ,L|\theta \right)$ was transformed via the inverse FFT to yield the time-domain signal $\hat{P}\left(t,L|\theta \;\right)$.

It is well-known that PTT is the most critical high-sensitivity parameter in the uniform TL model (Sugimachi et al., 2001; Fazeli et al., 2014). Our parametric sensitivity analysis of the tapered tube-load model indicated that PTT is likewise the most critical high-sensitivity parameter in the tapered TL model. Hence, the TL model fitting problem in (3) was solved by (i) sweeping θ_2_ over a physiologically plausible range while (ii) θ_1_ and θ_3_ were determined for each value of θ_2_. In deriving the optimal θ_1_ and θ_3_ associated with each θ_2_, multiple (85) initial guesses were employed to ensure that the solution obtained from the optimization problem corresponds to (or at least is very close to) global minimum. For each θ associated with each of the θ_2_ values examined, the cost function in Equation (3) was evaluated. Then, θ associated with the minimum cost function value was determined as θ^*^. In this way, the integrity and accuracy of the estimated TL model parameters was maximized.

The validity of the TL models was then assessed using both testing and training data. The testing data were employed to assess (i) the goodness of fit including the root-mean-squared error (RMSE) and correlation coefficient (*r* value) between the measured aortic outlet BP waveform vs. model-predicted aortic outlet BP waveform derived from the aortic inlet BP waveform as well as (ii) the accuracy-complexity trade-off via the Akaike Information Criterion (AIC), and also (iii) the goodness of fit including the root-mean-squared error (RMSE) and correlation coefficient (*r* value) between the measured aortic inlet BP waveform vs. model-predicted aortic inlet BP waveform derived from the aortic outlet BP waveform (to assess the ability of the two TL models as the basis for estimating central from distal BP). The AIC was computed as follows:

(5) $$\begin{array}{r} AIC=N\ln\frac{1}{N}\sum_{t=1}^{N}{\left[P\left(t,0\right)-\hat{P}\left(t,0|\theta^{*}\right)\right]}^{2}+2K\\ \;+\frac{2K\left(K+1\right)}{N-K-1} \end{array}$$

where *N* is the size of the testing data and *K* is the number of model parameters. The first term rewards the goodness of fit, while the second term penalizes the complexity. The third term is intended to compensate for the limited size of the testing data (Burnham and Anderson, 2003). These metrics were first computed for both the tapered and uniform TL models on the subject-by-subject basis and then were aggregated across all subjects for comparison. For the RMSE and r value, mean and standard deviation (SD) were computed. The statistical significance in the difference in these metrics was determined using the Wilcoxon signed-rank test with the Holm-Bonferroni correction for multiple comparisons. For the AIC, the tapered vs. uniform TL models were compared in terms of the number of subjects in which the AIC metric was smaller (note that smaller AIC implies superior accuracy-complexity trade-off). The training data were employed to assess (i) the frequency response of the two TL models in comparison to the data-based non-parametric frequency response in individual subjects as well as (ii) the physiological relevance of PTT values associated with the two TL models in comparison to the PTT derived directly from the aortic inlet and outlet BP waveforms using the intersecting tangent method (Gaddum et al., 2013).

## Results

**Figure 2:**
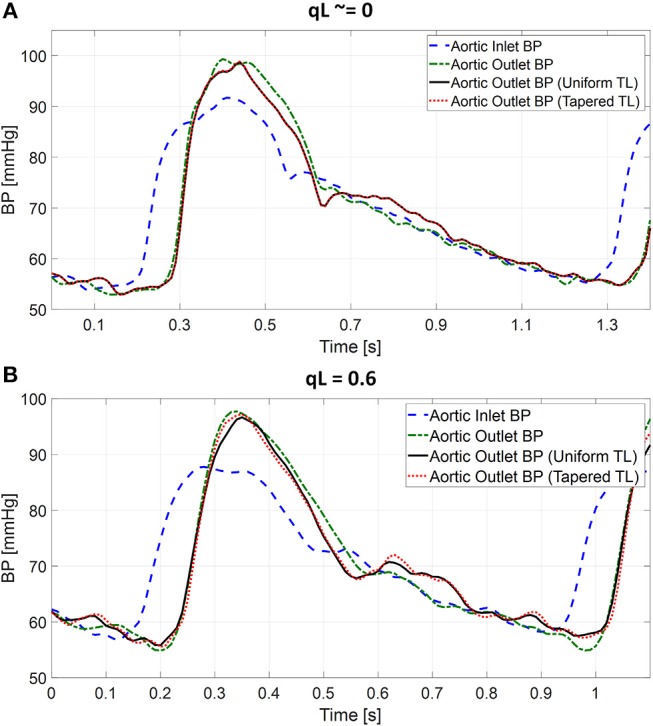
Representative femoral blood pressure (BP) waveforms derived from tapered vs. uniform tube-load (TL) models when ascending aortic BP waveform was inputted. **(A)** Uniform TL model was preferred in terms of AIC (*qL*≅0). **(B)** Tapered TL model was preferred in terms of AIC (*qL* = 0.6).

**Figure 3:**
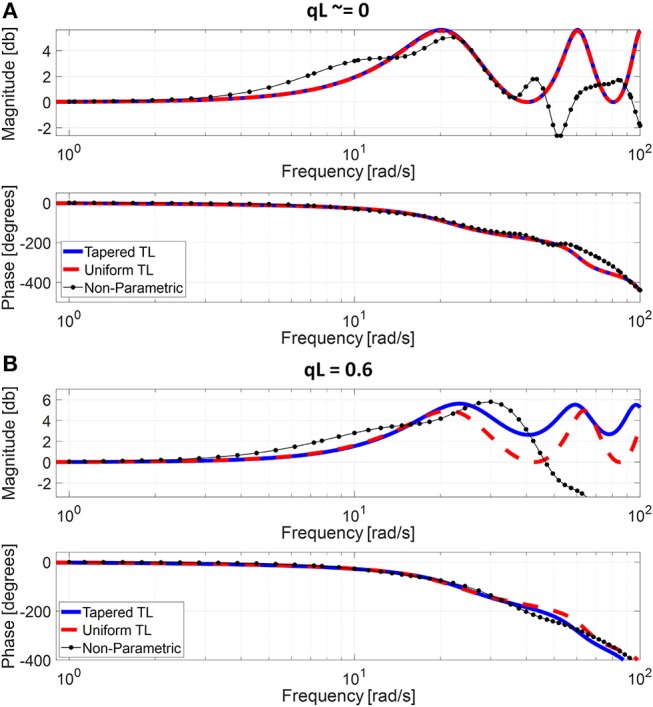
Representative frequency responses of the two tube-load (TL) models in comparison with the non-parametric frequency response derived directly from the aortic inlet and outlet blood pressure (BP) waveforms. **(A)** Uniform TL model was preferred in terms of AIC (*qL*≅0). **(B)** Tapered TL model was preferred in terms of AIC (*qL* = 0.6).

**Figure 4:**
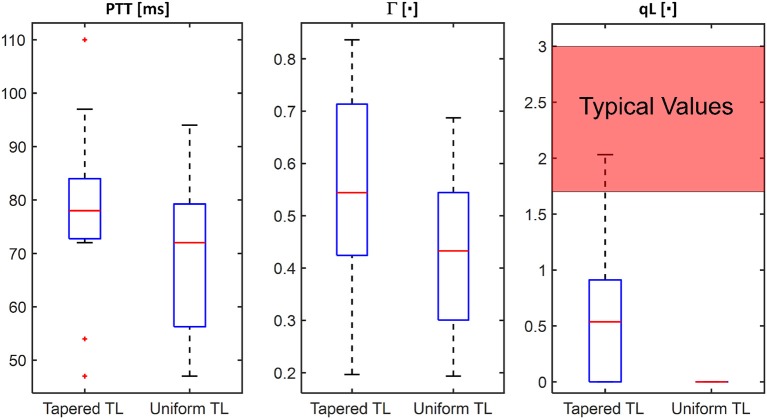
Comparison of individual-specific pulse transit time (PTT), reflection constant (Γ), and radius ratio (qL) values associated with tapered vs. uniform tube-load (TL) models.

**Figure 5:**
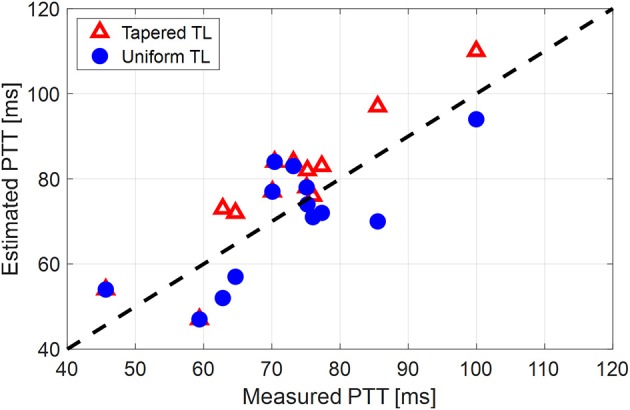
Individual-specific pulse transit time (PTT) values associated with the two tube-load (TL) models in comparison to the PTT values derived directly from the aortic inlet and outlet blood pressure (BP) waveforms.

Table 1 summarizes the validity metrics of the tapered vs. uniform TL models, including the RMSE, correlation coefficient (*r* value), and the number of preferred TL model (tapered vs. uniform) in terms of the AIC values. Figure 2 shows representative aortic outlet (femoral) BP waveforms derived from the two TL models associated with (a) θ_1_ = *qL*≅0 and (b) θ_1_ = *qL* = 0.6 when aortic inlet (central aortic) BP waveform was inputted. Figure 3 shows representative frequency responses of the two TL models associated with (a) θ_1_ = *qL*≅0 and (b) θ_1_ = *qL* = 0.6 in comparison with the non-parametric frequency response derived directly from the aortic inlet and outlet BP waveforms. Table 2 summarizes the parameter values estimated for the two TL models. Figure 4 compares the individual-specific PTT and reflection constant values between the two TL models. Figure 5 shows the individual-specific PTT values associated with the two TL models in comparison to the PTT values derived directly from the aortic inlet and outlet BP waveforms.

**Table 1:** The validity metrics of the tapered vs. uniform TL models, including the root-mean-squared error (RMSE), correlation coefficient (*r* value), and the number of TL models associated with the smallest Akaike Information Criterion (AIC) values.

	**RMSE [mmHg]**		**Correlation Coefficient**		**AIC**
	**Outlet BP**	**Inlet BP**	**Outlet BP**	**Inlet BP**	**Outlet BP**
Tapered TL	3.3+/−1.1	2.5+/−1.1	0.98+/−0.02	0.98+/−0.01	6
Tapered TL (1.7 ≤ q ≤ 3)	3.9+/−1.1^*^	2.8+/−1.1^*^	0.97+/−0.02^*^^†^	0.98+/−0.01^*^	0
Uniform TL	3.4+/−1.1	2.8+/−1.0^*^	0.98+/−0.01	0.98+/−0.01^*^	7

* *Significantly different from tapered TL (p <0.05 with Holm-Bonferroni correction)*.

† *Significantly different from uniform TL (p <0.05 with Holm-Bonferroni correction)*.

**Table 2:** Parameter values estimated for the two TL models.

	**PTT (τ) [ms]**	**Reflection Constant (Γ)**	**Tapering Constant (qL)**
Tapered TL	78+/−16	0.55+/−0.19	0.6+/−0.7
Tapered TL (1.7 ≤ q ≤ 3)	84+/−02^†^	0.73+/−0.09^*^^†^	1.7+/−0.1^*^^†^
Uniform TL	70+/−13^*^	0.43+/−0.15^*^	0^*^

* *Significantly different from tapered TL (p <0.05 with Holm-Bonferroni correction)*.

† *Significantly different from uniform TL (p <0.05 with Holm-Bonferroni correction)*.

## Discussion

The uniform TL model has the potential to enable patient-specific assessment of CV health with its minimal number of physiologically interpretable model parameters that may be individualized using small amount of data. Despite its demonstrated success in CV health and disease monitoring applications, opportunities exist for its potential improvement by incorporating realistic components. In this study, the effect of adding an exponential tapering to the TL model as an approximation for aortic geometric tapering on its predictive performance and physiological relevance was investigated.

The exponentially tapered and uniform TL models exhibited comparable goodness of fit for the aortic outlet BP whose differences were not statistically significant, both in terms of RMSE and correlation coefficient (Table 1 and Figure 2). The two TL models also exhibited frequency responses comparable to the non-parametric frequency response derived from the aortic and femoral BP waveforms in most patients (Figure 3; note that the non-parametric frequency response may not be meaningful beyond ~30 rad/s considering that the energy associated with the arterial BP waveform is concentrated in the heart rate and its 3~4 harmonics and that the heart rate of the subject is ~1 Hz, Figure 2). As a consequence, the uniform TL model was superior to the exponentially tapered TL model in terms of AIC by virtue of its smaller number of requisite parameters (uniform: 2; tapered: 3): the former was preferred to the latter in 7 out of 13 subjects (Table 1). Although the number of subjects in which the uniform TL model was preferred to its tapered counterpart was not dominant, the difference in the goodness of fit between the two TL models was small even in the remaining 6 subjects in which the tapered TL model was preferred to its uniform counterpart (<3% on the average in terms of RMSE; see Figure 2B). Further, the frequency responses were quite comparable in the 6 subjects in which the tapered TL model was preferred to its uniform counterpart (see Figure 3B). On the other hand, there was a notable difference between the two TL models in only 2 subjects. Hence, it may be argued that the uniform TL model is a viable model to represent the BP wave propagation in the aorta, and that the benefit of using the exponentially tapered TL model may not be large. As an additional note, the goodness of fit for the aortic inlet BP associated with the same TL models showed that the tapered TL model was significantly superior to its uniform counterpart both in terms of RMSE and correlation coefficient. Yet, the absolute amount of difference was only marginal.

**Figure 6:**
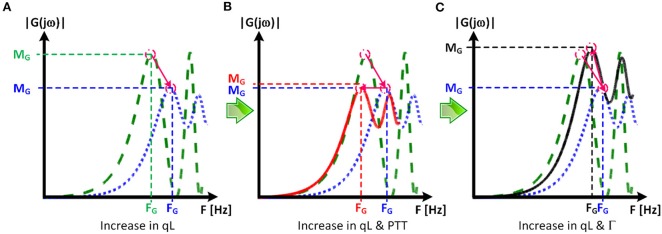
Parametric sensitivity of the frequency response associated with the tapered tube-load (TL) model. **(A)** An increase in the tapering constant (qL) decreases the amplitude M_G_ of the first peak in the frequency response while increasing its frequency coordinate F_G_. Green dashed line: frequency response of uniform TL model. Blue dotted line: frequency response of tapered TL model (qL > 0) with pulse transit time (PTT) and reflection constant (Γ) identical to uniform TL model. **(B)** An increase in the PTT in the tapered TL model decreases F_G_. Red solid line: frequency response of tapered TL model in **(A)** with larger PTT. **(C)** An increase in the reflection constant in the tapered TL model increases M_G_ while decreasing F_G_. Black solid line: frequency response of tapered TL model in **(A)** with larger reflection constant.

The comparable goodness of fit between the exponentially tapered and uniform TL models was supported by the values of the tapering constant (θ_1_ = *qL*) estimated for the exponentially tapered TL model: it was estimated to be 0.6 on the average, which corresponds to the aortic inlet-outlet radius ratio of 1.5. In addition, its value exhibited a large degree of inter-individual variability (1.17 in terms of the coefficient of variation). Interestingly, the tapering constant was very close to zero (corresponding to zero tapering and aortic inlet-outlet radius ratio of 1) in 6 out of the 13 subjects investigated in this study (including the subject shown in Figure 2A), while in no subjects its value was large enough to be compatible with the anatomically plausible typical value associated with the femoral artery (~2.5, which corresponds to the aortic inlet-outlet radius ratio of ~3.5 Wang and Parker, 2004; Matthys et al., 2007; Reymond et al., 2009). Despite its relatively small value, the aortic tapering also impacted the remaining parameters in the TL model. In particular, the PTT values derived from the fitting associated with the uniform TL model were comparable to the PTT measured directly from the aortic inlet and outlet BP waveforms (72+/−12 [ms]), whereas the PTT values derived from the fitting associated with the tapered TL model were significantly different from the PTT measured directly from the data (Table 2 and Figures 4, 5). In fact, the tapered TL model exhibited a tendency of overestimating the PTT relative to the direct PTT measurements across all subjects (Figure 5). In addition, the tapered TL model had significantly larger reflection constants than the uniform TL model (Table 2). These trends can be explained by analyzing the parametric sensitivity of the frequency response associated with the tapered TL model [especially, the amplitude (M_G_) and location (F_G_) of its first peak, which may be the most practically critical peak considering the limited frequency contents of the aortic BP signals (Hahn et al., 2009); Figure 6]. First, an increase in the tapering constant decreases the amplitude M_G_ of the first peak in the frequency response while increasing its frequency coordinate F_G_ (Figure 6A). Second, an increase in the PTT in the tapered TL model decreases F_G_ (Figure 6B). Third, an increase in the reflection constant in the tapered TL model increases M_G_ while decreasing F_G_ (Figure 6C). Putting them altogether, the presence of aortic tapering in the tapered TL model is compensated for by the larger PTT and reflection constant relative to the uniform TL model (i.e., tapered TL model with zero aortic tapering), in such a way that the amplitude (M_G_) and location (F_G_) of the first peak in the frequency response associated with the two TL model remain the same as dictated by the data to be fitted.

When the tapering constant was constrained in solving the model fitting problem in Equation (3) to restrict the aortic inlet-outlet radius ratio in the vicinity of its anatomically plausible value (1.7~3.0), the exponentially tapered TL model underperformed the uniform TL model both in terms of RMSE and correlation coefficient (Table 1). Interestingly, the tapering constant was estimated to be its lower bound in most subjects (11 out of 13), which suggests that the exponentially tapered TL model tends to somehow minimize the degree of aortic tapering in order to maximize its goodness of fit. In addition, perhaps due to the restrictions imposed on the tapering constant, the values of PTT and reflection constant were significantly different from those associated with the uniform TL model (Table 2). It is noted that both PTT and reflection constant were significantly over-estimated relative to the uniform TL model, consistently with the insight obtained from the parametric sensitivity analysis in Figure 6.

**Figure 7:**
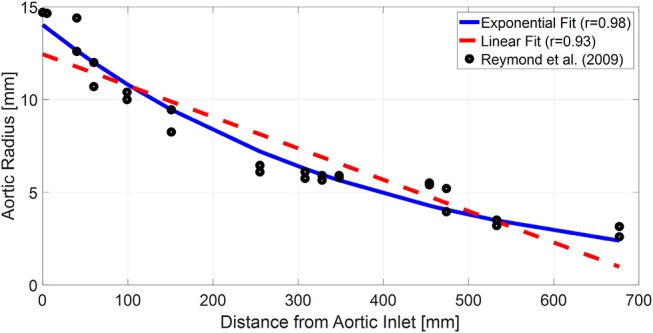
Typical anatomical aortic diameter data with respect to the distance from aortic inlet (black circles) and its exponential (blue solid line) and linear (red dashed line) fits. The anatomical data are from a prior work (Reymond et al., 2009).

On the one hand, the results all in all suggest that exponential aortic tapering may not be physiologically relevant for at least two reasons. First, the TL model with exponential tapering tends to fall back to the uniform TL model as it is fitted to the experimental data. Second, the TL model with exponential aortic tapering exhibited poor predictive accuracy than the uniform TL model if anatomically plausible aortic tapering was enforced. In fact, this finding may be corroborated by a prior study, which showed that the tapered TL model did not exhibit superiority to its uniform counterpart in representing the aortic impedance (Fogliardi et al., 1997). On the other hand, the results may not be simply interpreted as there being no value in incorporating the aortic tapering into TL models in general. In fact, available anatomical data on the aortic radius with respect to the distance from the heart (Wang and Parker, 2004; Matthys et al., 2007; Reymond et al., 2009) could be fitted reasonably well with an exponential function (*r* > 0.97; Figure 7). In addition, aortic tapering is known to be associated with continuous wave reflection (Segers and Verdonck, 2000) as well as the distribution of atherosclerosis (Sundell and Roach, 1998). One plausible explanation for the limited benefit of incorporating aortic tapering in the TL model is that the aortic tapering estimated for the tapered TL model considered in this work includes the influence of aortic branching, which was not explicitly modeled in this work but is known to offset the aortic tapering to yield impedance matching in the forward direction (Noordergraaf, 1978). In particular, considering that femoral BP was used as aortic outlet BP in this work and also that aortic branching rather than geometric tapering may be the primary factor responsible for the reduction of arterial diameter beyond the abdominal aorta, it may not be trivial to interpret the tapering constant estimated in this work. In this regard, it may be worth investigating how to appropriately incorporate both the aortic tapering and branching in order to enhance the TL models in terms of both goodness of fit and physiological relevance.

## Conclusion

The results of this study suggest that the uniform TL model may be more robust and thus preferred as representation for BP wave propagation in human aorta relative to the exponentially tapered TL model. In comparison with the uniform TL model, the exponentially tapered TL model may not provide valid physiological insight on the aortic tapering, and the improvement in the goodness of fit offered by the exponential aortic tapering may only be marginal. Considering that exponential aortic tapering is relevant from physiological standpoint, future work on more rigorous investigation and refinement of exponentially tapered TL model will be rewarding.

## Data Availability

The datasets for this manuscript are not publicly available because no IRB approval and participant consent were obtained for making the data publicly available. Requests to access the datasets should be directed to J-OH, jhahn12@umd.edu.

## Ethics Statement

The study was carried out in accordance with the recommendations of the University of Alberta Health Research Ethics Board with written informed consent from all subjects. All subjects gave written informed consent in accordance with the Declaration of Helsinki. The protocol was approved by the University of Alberta Health Research Ethics Board (ID Pro00021889).

## Author Contributions

J-OH and RM designed the study. BF and MM conducted human subject study. AM, AT, RM, and J-OH analyzed the data. BF, MM, RM, and J-OH reviewed the data analysis results. AM and J-OH drafted and revised the manuscript. AT, BF, MM, and RM reviewed the manuscript.

### Conflict of Interest Statement

The authors declare that the research was conducted in the absence of any commercial or financial relationships that could be construed as a potential conflict of interest.

## Appendix

### Exponentially Tapered Tube-Load Model

In this study, a variant of the exponentially tapered tube-load model of the aorta developed by Fogliardi et al. (1997) was used. This model is explained in this **Appendix**. If the radius of the aorta is assumed to decrease with the exponential rate of $\frac{q}{2}$ with respect to the distance *x* from the inlet (i.e., the aortic valve):

(A.1) $$\begin{array}{r} r\left(x\right)=r_{0}e^{-\frac{q}{2}x} \end{array}$$

where *r*_0_ is the aortic inlet radius, then the aortic inertance, compliance, and characteristic impedance can be expressed as follows, according to the quadratic dependence of the aortic inertance and compliance on the radius based on the assumptions that (i) the aortic incremental Young modulus is constant and (ii) the aortic wall thickness is proportional to the aortic radius:

(A.2) $$\begin{array}{r} l\left(x\right)=l_{0}e^{qx},\;c\left(x\right)=c_{0}e^{-qx},\;Z_{c}\left(x\right)=\sqrt{\frac{l\left(x\right)}{c\left(x\right)}}=Z_{c0}e^{qx} \end{array}$$

where *l*(*x*), *c*(*x*), and *Z*_*c*_(*x*) are the aortic inertance, compliance, and characteristic impedance, while *l*_0_, *c*_0_, and *Z*_*c*0_ are their respective values at the aortic inlet.

The BP wave at a distance *x* from the aortic inlet is given by the sum of the forward-traveling and backward-traveling BP waves:

(A.3) $$\begin{array}{r} P\left(j\omega ,x\right)=P_{f}\left(j\omega ,0\right)e^{-\left(\Delta -\frac{q}{2}\right)x}+P_{b}\left(j\omega ,0\right)e^{\left(\Delta +\frac{q}{2}\right)x} \end{array}$$

where *P*_*f*_(*jω*, 0) and *P*_*b*_(*jω*, 0) are the forward-traveling and backward-traveling BP waves at the aortic inlet, ω is frequency, *j* is the imaginary unit (*j*^2^ = −1), and $\Delta =\sqrt{\gamma^{2}+{\left(\frac{q}{2}\right)}^{2}}$ is the transfer constant with $\gamma =j\omega \sqrt{l\left(x\right)c\left(x\right)}=j\omega \sqrt{l_{0}c_{0}}$ the propagation constant. The forward-traveling and backward-traveling BP waves *P*_*f*_(*jω*, 0) and *P*_*b*_(*jω*, 0) are related to each other by the wave reflection constant Γ:

(A.4) $$\begin{array}{r} \Gamma \left(j\omega ,0\right)=\frac{P_{b}\left(j\omega ,0\right)}{P_{f}\left(j\omega ,0\right)}=\frac{\frac{R_{p}}{\gamma }\left(\Delta -\frac{q}{2}\right)-Z_{c0}e^{qL}}{\frac{R_{p}}{\gamma }\left(\Delta +\frac{q}{2}\right)+Z_{c0}e^{qL}}e^{-2\Delta L} \end{array}$$

where *L* is the distance between the aortic inlet and outlet. Combining (A.3) and (A.4), the relationship between the aortic inlet and outlet BP waves is expressed as follows:

(A.5) $$\begin{array}{l} \frac{P\left(j\omega ,L\right)}{P\left(j\omega ,0\right)}=\frac{e^{-\left(\Delta -\frac{q}{2}\right)L}+\Gamma \left(j\omega ,0\right)e^{\left(\Delta +\frac{q}{2}\right)L}}{1+\Gamma \left(j\omega ,0\right)}\;\\ \;=\frac{2\frac{R_{p}}{\gamma }\Delta e^{-\left(\Delta -\frac{q}{2}\right)L}}{\frac{R_{p}}{\gamma }\left(\Delta +\frac{q}{2}\right)+Z_{c0}e^{qL}+\left[\frac{R_{p}}{\gamma }\left(\Delta -\frac{q}{2}\right)-Z_{c0}e^{qL}\right]e^{-2\Delta L}} \end{array}$$

The relationship (A.5) can be parameterized with θ_1_ = *qL*, $\theta_{2}=\tau =L\sqrt{l_{0}c_{0}}$, and $\theta_{3}=\frac{R_{p}}{Z_{c0}}$ as follows:

(A.6) $$\begin{array}{r} \frac{P\left(j\omega ,L\right)}{P\left(j\omega ,0\right)}=\frac{2\theta_{3}\bar{\Delta }e^{-\left[\bar{\Delta }-\frac{\theta_{1}}{2}\right]}}{\theta_{3}\left(\bar{\Delta }+\frac{\theta_{1}}{2}\right)+j\omega \theta_{2}e^{\theta_{1}}+[\theta_{3}\left(\bar{\Delta }-\frac{\theta_{1}}{2}\right)-j\omega \theta_{2}e^{\theta_{1}}e^{]-2\bar{\Delta }}} \end{array}$$

where $\bar{\Delta }=\Delta L=\bar{\Delta }\left(\theta_{1},\theta_{2}\right)=\sqrt{{\left(\frac{\theta_{1}}{2}\right)}^{2}+{\left(j\omega \theta_{2}\right)}^{2}}$.
